# Gallstone as a cause of intestinal obstruction (Bouveret syndrome)

**DOI:** 10.1093/jscr/rjad582

**Published:** 2023-10-31

**Authors:** Emerson Leonardo Monteiro, Johannes Schmid, Hans Jörg Mischinger, Robert Sucher, Peter Kornprat

**Affiliations:** Department of General, Visceral and Transplantation Surgery, Medical University of Graz, Graz 8036, Austria; Division of General Radiology, Department of Radiology, Medical University of Graz, Graz 8036, Austria; Department of General, Visceral and Transplantation Surgery, Medical University of Graz, Graz 8036, Austria; Department of General, Visceral and Transplantation Surgery, Medical University of Graz, Graz 8036, Austria; Department of General, Visceral and Transplantation Surgery, Medical University of Graz, Graz 8036, Austria

**Keywords:** ileus, gallstone disease, Bouveret syndrome, Mirizzi syndrome

## Abstract

Gallstone ileus is a rare cause of bowel obstruction. Here we report about two cases with clinical findings and therapy options. Both patients were presented with typical ileus-like symptoms, although the surgical treatment differs due to the CT scan and intraoperative findings. There are many methods for treating patients with Bouveret syndrome. Endoscopy should be the first treatment option for young patients with no significant diseases in the medical history, depending on the size of the stone. Surgical approach is the next possible option. Combination of these two methods is associated with higher mortality. In case there is no extraluminal gas or intraperitoneal fluid in CT-scan, there is no need for an acute surgery. Conservative therapy prior to the intervention enables a precise planning of whether the endoscopic approach or open surgery would be beneficial for the patient.

## Introduction

Gallstone disease is the leading cause of hospitalization in gastroenterology, affecting up to 20% of the European population [1]. Solely the presence of a stone in the gallbladder does not fulfill the criteria for gallstone disease. These stones must be symptomatic or cause any type of complication [2]. One of the rare complications of gallstones apart from the well-known dyspeptic disorder or pain can be intestinal obstruction occurring as a cause in 1–3% [3]. Here, we present two cases of an ileus caused by gallstones.

## Case 1

**Figure 1:**
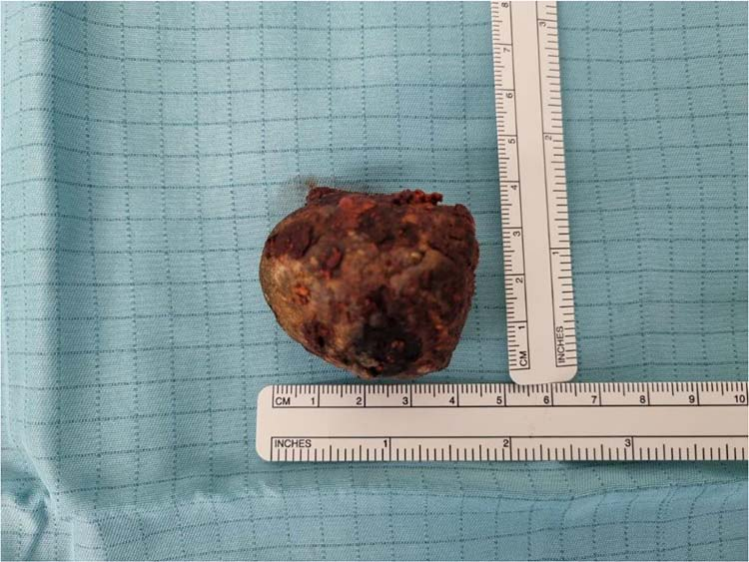
Picture of the gallstone.

**Figure 2:**
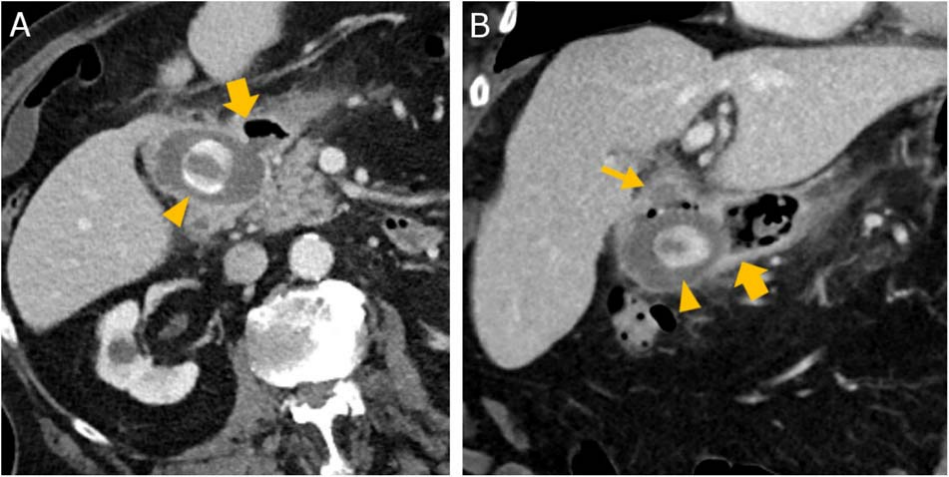
Abdominal CT in axial (A) and oblique paracoronal reconstruction (B); gallstone in the duodenal bulb (arrowhead  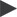), pylorus (thick arrow  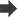), chronically inflamed, and collapsed gall bladder with fistula to the duodenum (thin arrow  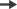); image courtesy of Gerald Wolf, State Hospital Western Styria, Austria.

A 79-year-old male patient was referred from a peripheral hospital with an intestinal obstruction caused by a gallstone. Previous endoscopy showed a penetration of the gallstone through the wall of the gallbladder into the lumen of the duodenum (Fig. 1—endo bild). Due to its enormous size, the stone prevented bowel transit and the patient presented the typical ileus symptoms such as nausea, vomiting, and abdominal pain. After placement of a nasogastric tube, the symptoms noticeably improved. In the abdominal computed tomography (CT)-scan, both extraluminal gas and intraperitoneal fluid were excluded (Fig. 2) and under antibiotic treatment, the levels of inflammatory markers decreased. The stable medical state allowed primary conservative therapy prior to the surgery.

During the open procedure, the gallbladder was separated from the duodenum and removed. As a consequence of chronical inflammatory changes, the remaining common bile duct could not be identified with certainty, so intraoperative cholangiography clarified the difficult anatomy and excluded another bile stone in the biliary tree. The perforation of the duodenum was primarily closed with interrupted sutures in two layers with an additional Graham patch.

In the postoperative course, a sudden drop in hemoglobin levels occurred and melena was found, indicating upper gastrointestinal bleeding. Due to the previous surgery and symptoms, the most probable origin of bleeding was the duodenum. The bleeding was endoscopically confirmed and stopped with Hemospray. After several days of observation, the patient was transferred back to the peripheral hospital.

## Case 2

**Figure 3:**
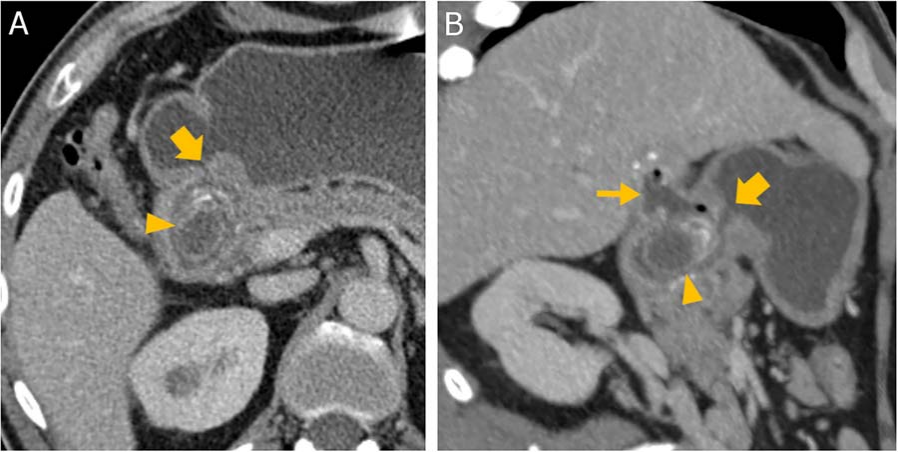
Abdominal CT in axial (A) and oblique parasagittal reconstruction (B); gallstone in the duodenal bulb (arrowhead  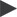), pylorus (thick arrow  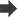), chronically inflamed, and collapsed gall bladder with fistula to the duodenum (thin arrow   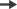); image courtesy of Helmut Schöllnast, State Hospital Graz II, Austria.

A 57-year-old male patient was also referred from a peripheral hospital with a history of vomiting and abdominal pain, with the CT-scan showing Bouveret syndrome (Fig. 3). Similar to the first patient, due to the stable condition, conservative treatment could precede surgical intervention.

The local findings and cholangiography confirmed, in addition to Bouveret syndrome, a typical presentation of Mirizzi syndrome Grade III.

The duodenal perforation was again closed with simple interrupted sutures in two layers. Unlike the first patient, the common bile duct was damaged due to inflammation and therefore reconstructed with hepatojejunostomy with temporal extraperitoneal biliary drainage. The drainage was removed in 6 weeks during follow-up gastroscopy.

## Discussion

There are multiple treatment options for Bouveret syndrome. In young patients with no significant diseases in medical history, the first choice is an endoscopic approach, such as endoscopic extraction or endoscopic lithotripsy, depending on the size of the stone. A drawback of the endoscopic methods is, for example, that with lithotripsy, the fragments could again cause gallstone ileus in distal parts of the intestinal tract or with endoscopic extraction, the procedure is limited by the stone size.

The other option is open surgery ranging from a simple duodenotomy to Whipple procedure. If the patient is in a bad condition or the endoscopic methods seem not to be possible or sufficient, open surgery should be the first choice. Combination of endoscopy and open surgery has been associated with a higher mortality of 20–30% compared to the simple duodenotomy [4].

The third therapy option is extracorporeal shockwave lithotripsy. This method requires multiple sessions and is always additionally combined with endoscopy.

Both patients presented in this case report were right away indicated for open surgery based on the size of the stone in CT-scan. As mentioned, the combination of endoscopy and surgery is associated with higher mortality, and the first patient was already suffering from many chronical diseases that increase the mortality. Adding endoscopic treatment before the surgery would probably lead to prolonged hospitalization.

## Conclusion

These two cases show that there is no need to perform an acute surgery when patients are in a stable medical state and the CT-scan shows no trace of free intraperitoneal fluid or extraluminal air. Conservative treatment could buy some time to precisely plan the surgery, other treatment options, or transport the patient to a central hospital with more experience with such a complication of cholecystolithiasis.

The method of treatment should be chosen based on the medical condition of the patient or on the experience of the medical center with the therapeutic options to achieve the best outcome.

## Data Availability

The data that support the findings of the study are available on request from the corresponding author.

## References

[1] Gutt C, Schläfer S, Lammert F. The treatment of gallstone disease. Dtsch Arztebl Int 2020;117:148–58. 32234195 10.3238/arztebl.2020.0148 PMC7132079

[2] Lammert F, Gurusamy K, Ko CW, et al. Gallstones. Nat Rev Dis Primers 2016;2:16024. 27121416 10.1038/nrdp.2016.24

[3] Morales-Ortiz JA, Cota-Novoa MM, Bernal Mora GF, et al. Intestinal obstruction secundary to gallstone ileus: case report. Cir Cir 2021;89:31–3. 34932536 10.24875/CIRU.21000022

[4] Doycheva I, Limaye A, Suman A, et al. Bouveret's syndrome: case report and review of the literature. Gastroenterol Res Pract 2009;2009:914951. 10.1155/2009/914951. 19360112 PMC2666152

